# Prognostic Value of Circular RNA ciRS-7 in Various Cancers: A PRISMA-Compliant Meta-Analysis

**DOI:** 10.1155/2020/1487609

**Published:** 2020-01-22

**Authors:** Guangwei Tian, Guang Li, Lin Guan, Zihui Wang, Nan Li

**Affiliations:** ^1^Department of Radiation Oncology, The First Affiliated Hospital of China Medical University, Shenyang 110001, China; ^2^Department of Gastrointestinal, The First Affiliated Hospital of China Medical University, Shenyang 110001, China; ^3^Department of Neuroscience, Cleveland Clinic, Cleveland, OH 44106, USA

## Abstract

**Background:**

Circular RNAs (circRNAs) have been shown to be involved in tumorigenesis. As a member of circRNAs, ciRS-7 is thought to be a negative prognostic indicator in multiple types of cancer. The present study aimed to comprehensively explore the value of ciRS-7 in tumor malignancy. *Materials and Methods*. A systematic review of PubMed, Web of Science, and the Cochrane library was carried out to examine the related studies. The pooled odds ratios (ORs) and hazard ratios (HRs) with 95% confidence intervals (95% CIs) were calculated from the available publications by STATA 12.0. Subgroup analysis, publication bias, sensitivity analysis, and meta-regression were conducted.

**Results:**

This meta-analysis included 1,714 patients from 13 cohorts. The results suggested that high ciRS-7 expression was significantly associated with overall survival (OS) (HR = 2.17, 95% CI = 1.50–3.15, *P* < 0.001) in various cancers. Stratified analyses indicated that elevated levels of ciRS-7 appeared to be a powerful prognostic biomarker for patients with non-small-cell lung cancer (NSCLC) (HR: 2.50, 95% CI: 1.07–6.07, *P* < 0.001) in various cancers. Stratified analyses indicated that elevated levels of ciRS-7 appeared to be a powerful prognostic biomarker for patients with non-small-cell lung cancer (NSCLC) (HR: 2.50, 95% CI: 1.07–6.07, *P* < 0.001) in various cancers. Stratified analyses indicated that elevated levels of ciRS-7 appeared to be a powerful prognostic biomarker for patients with non-small-cell lung cancer (NSCLC) (HR: 2.50, 95% CI: 1.07–6.07, *P* < 0.001) in various cancers. Stratified analyses indicated that elevated levels of ciRS-7 appeared to be a powerful prognostic biomarker for patients with non-small-cell lung cancer (NSCLC) (HR: 2.50, 95% CI: 1.07–6.07, *P* < 0.001) in various cancers. Stratified analyses indicated that elevated levels of ciRS-7 appeared to be a powerful prognostic biomarker for patients with non-small-cell lung cancer (NSCLC) (HR: 2.50, 95% CI: 1.07–6.07,

**Conclusions:**

High expression of ciRS-7 has a significant correlation with the high stage in various cancers, and ciRS-7 is intimately associated with an adverse OS in numerous cancers. Thus, ciRS-7 may act as a potential biomarker for the development of malignancies.

## 1. Introduction

Circular RNAs are a type of noncoding RNAs that have a unique single-stranded closed ring structure, which were discovered in viroid via electron microscopy as early as in 1976, and originally thought to be that with incorrect splicing of RNA [1, 2]. Researchers have been focusing on circRNAs since the application of high-throughput RNA sequencing and bioinformatics analyses, and thousands of human circRNAs have been identified so far [3, 4].

Recently, a lot of studies have confirmed a functional role played by circRNAs in cancer, which is circRNAs could be involved in tumor proliferation, angiogenesis [5], invasion, and metastasis [6]. Furthermore, evidence has showed that circRNAs could regulate most critical signaling pathways in cancer including Wnt [7], PIK3/AKT [8], and MAPK/ERK [9] pathways and deregulated expression of circRNAs in many types of cancers, including GC [10], bladder cancer [11], breast cancer [12], colorectal cancer [13], lung cancer [14], and so on.

ciRS-7, also known as the antisense to the cerebellar degeneration-related protein 1 transcript (CDR1as), is one of the circRNAs specifically binding to human microRNA-7 (miR-7) as a sponge [15, 16]. Currently, ciRS-7 has received increasing attention and became one of the most extensively studied circRNAs in cancer. In MiOncoCirc dataset, ciRS-7 (CDR1-AS) is classified into the ubiquitous category and expressed in 19 types of cancer [17]. The upregulated expression of ciRS-7 is found in several cancers and contributes to the development of several cancers, including non-small-cell lung cancer (NSCLC) [18], colorectal cancer (CRC) [19], and hepatocellular carcinoma (HCC) [20]. Besides, ciRS-7 participates in many critical cancer-related pathways, such as EGFR [21, 22], PTEN/PI3K/AKT [23], and NF-*κ*B [24, 25]. Furthermore, increasing evidences suggest that ciRS-7 is closely related to the prognosis and clinicopathological features of cancers [18, 19, 21–30]; however, the prognostic results remain inconsistent. Thus, we conducted this meta-analysis to achieve a precise evaluation of the prognostic value of ciRS-7 in cancer patients.

## 2. Materials and Methods

This meta-analysis was performed by following the PRISMA guidelines [31]. Systematic literature research was independently performed by two authors through electronic databases, including PubMed, Web of Science, and the Cochrane Library for eligible studies published until March 18, 2019. Studies were selected using the following terms: “CDR1-AS” or “cdr1as” or “ciRS-7” and “tumor” or “cancer” or “carcinoma”. Citation lists of review articles were manually searched for finding potentially eligible studies.

### 2.1. Study Selection

Two authors (Guangwei Tian and Lin Guan) independently evaluated all of the included studies, and any disagreement was resolved by consultation with the supervisor (Nan Li). The following inclusion criteria were considered: (a) studies investigating on patients with any type of cancers; (b) studies in which the patients were grouped by the expression of ciRS-7 via validating techniques; (c) studies reporting the sufficient information association between ciRS-7 expression and survival, in which the hazard ratio (HR) with 95% confidence interval (95% CI); (d) studies reporting the relationship between ciRS-7 expression and clinical stage of cancer patients. Exclusion criteria were as follows: (a) studies without sufficient or usable data; (b) duplicative publications; (c) reviews, case reports, editorials, and conference abstracts; (d) laboratory studies only investigating the molecular function of ciRS-7.

### 2.2. Data Extraction and Quality Assessment

The primary outcome used in this meta-analysis was overall survival. The secondary outcome was clinical stage. The following information was extracted from each study: first author name, year, country, tumor type, sample size and type, expression of ciRS-7, detection technique, follow-up duration, and HRs of ciRS-7 expression for overall survival (OS) with 95% CIs. In all the articles included in our study, the expression of ciRS-7 was detected in tumor tissues. The Newcastle–Ottawa Score (NOS) quality assessment system adopted for case-control studies was utilized to examine the methodological quality of studies [32]. Included studies were scored according to three broad perspectives, selection, comparability, and exposure, through the star scoring method with a range of lowest quality (0 star) to the highest quality (9 stars). A score of ≥6 indicated high quality [33].

### 2.3. Statistical Analysis

Meta-analysis was performed using Stata 12.0 software (Stata, College Station, TX, USA). The prognostic value of ciRS-7 expression in patients with various types of cancers was analyzed by the pooled HR associated with the 95% CI. If the HR and 95% CI were not directly reported, we extracted them from Kaplan–Meier curves [34]. ORs were extracted for evaluating the association between the expression of ciRS-7 and tumor stage. Higgins *I*^2^ statistic was performed for evaluating the heterogeneity [35]. If heterogeneity was present (*I*^2^ > 50% or *P* < 0.05), a random-effect model was applied [36]; otherwise, a fixed effect model was used [37]. Publication bias was conducted by Begg's test [38]. Sensitivity was also performed to identify the effect of the individual study data on summary for HRs and ORs.

## 3. Results

### 3.1. Characteristics of Studies

**Figure 1:**
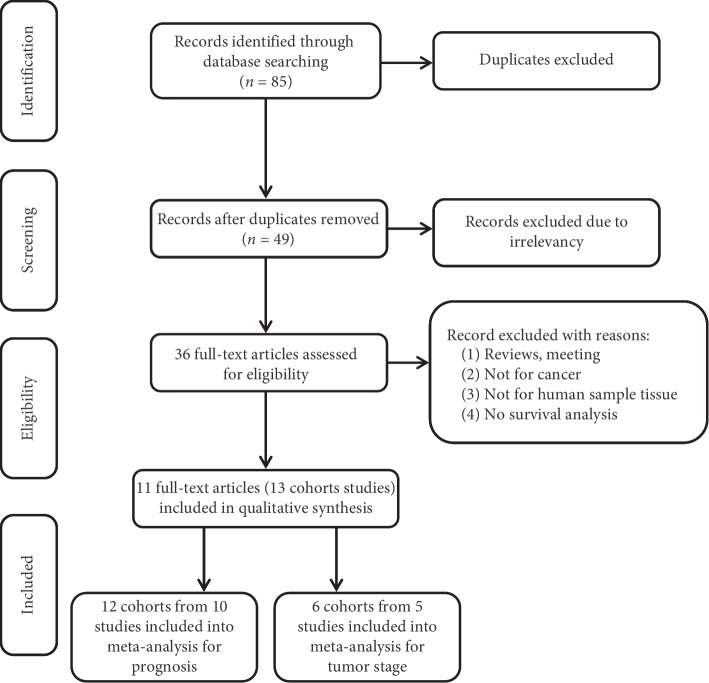
A flowchart of literature search and study selection.

There were 85 studies from a database search, and 74 irrelevant studies and duplicates were excluded after reviewing the titles and abstracts. On the basis of the inclusion and exclusion criteria, ultimately, 13 cohorts from 11 studies and 1,714 patients were included in this meta-analysis. A flowchart of the studies screening process is shown in Figure 1, and the detailed characteristics are summarized in Table 1. The enrolled studies were published in 2017 and 2018. The countries represented in the studies included China, Japan, and the Netherlands. Among these 13 cohorts, there were 7 types of cancers in the meta-analysis (3 NSCLC [18, 22, 24], 3 CRC [19, 21], 2 GC [23], 1 breast cancer [27], 2 esophageal squamous cell cancer (ESCC) [25, 29], 1 laryngeal squamous cell cancer (LSCC) [28], and 1 cholangiocarcinoma (CAA) [26]). ciRS-7 expression was measured in cancerous tissue by quantitative real-time PCR in all cohorts. Among 13 cohorts, 12 cohorts from 10 studies assessed the correlation between ciRS-7 and prognosis in various tumors [18, 19, 21–24, 26–29], and 6 cohorts from 5 studies reported the relationship between ciRS-7 and tumor stage [19, 22, 24, 29, 30]. HR and corresponding 95% CIs were extracted from the survival curve in one study. All included cohorts' NOS scores were ≥6 (Table 1).

### 3.2. Association between ciRS-7 and Overall Survival

**Figure 2:**
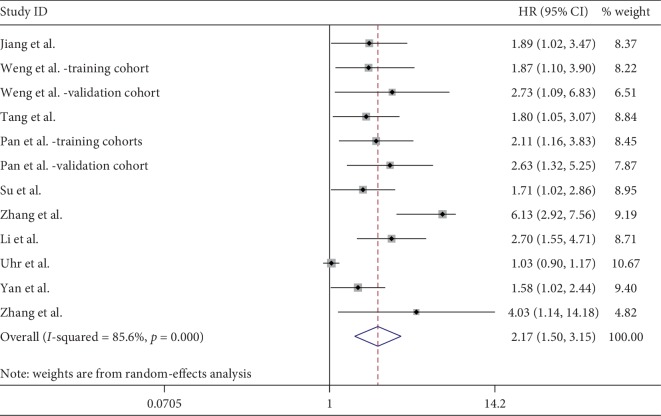
Forest plot for the association between ciRS-7 overall survival (OS) in various cancers.

In a pooled analysis of 12 cohorts, including 1,628 cancer patients [18, 19, 21–24, 26–29], the results indicated that high expression of ciRS-7 was significantly associated with poor OS (Figure 2). Due to a significant heterogeneity (*I*^2^ = 85.6%, *P* < 0.001), the random-effects model was used. The combined HR was 2.17 (95% CI: 1.50–3.15, *P* < 0.001). Next, a subgroup analysis was performed on cancer type and an elevated level of ciRS-7 was found to be associated with poor OS in NSCLC (HR: 2.50, 95% CI: 1.07–6.07, *P*=0.035), CRC (HR: 1.95, 95% CI: 1.34–2.84, *P* < 0.001), and GC (HR: 2.32, 95% CI: 1.48–3.64, *P* < 0.001). After stratified by ethnicity, we found an increased ciRS-7 which predicted a worse OS for Asians (HR: 2.35, 95% CI: 1.78–3.11, *P* < 0.001), but not for Caucasians (HR: 1.03, 95% CI: 0.90–1.17, *P*=0.659). When we regarded the sample size, analysis method, and different cutoff values, there was no significant change for pooled HR in each subgroup (Table 2). Due to significant heterogeneity in analysis, we detected the source of heterogeneity by multivariate meta-regression analysis. It was found that sample size (*P*=0.066), cancer type (*P*=0.860), ethnicity (*P*=0.111), analysis method (*P*=0.772), cutoff value (*P*=0.208), and study design (*P*=0.924) were not the sources of heterogeneity (Table 2).

**Figure 3:**
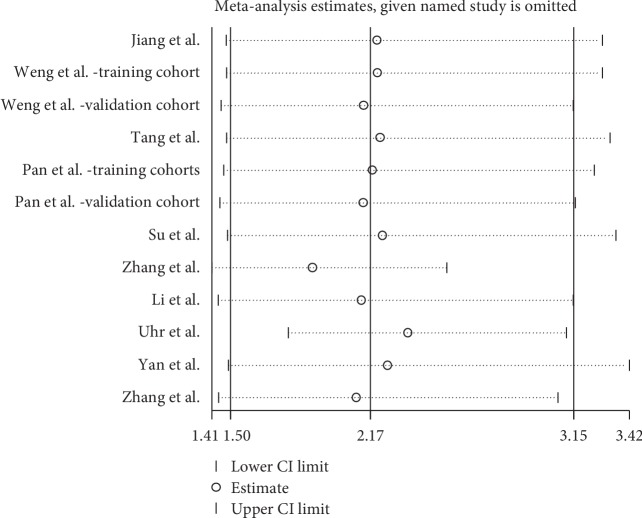
Sensitivity analysis between ciRS-7 expression and overall survival (OS).

**Figure 4:**
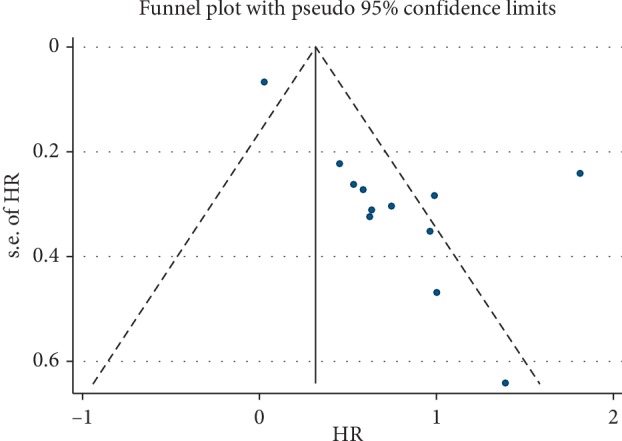
Publication bias of ciRS-7 expression and overall survival (OS).

A sensitivity analysis was conducted to assess whether one study would affect the overall results and to confirm the stability of results. In Figure 3, the results were relatively stable. The funnel plot was asymmetry (Figure 4). Additionally, Begg's test was used for quantitative analysis, and *P* value was 0.115, which indicated no publication bias in this meta-analysis.

### 3.3. Association between ciRS-7 and Clinical Stage

**Figure 5:**
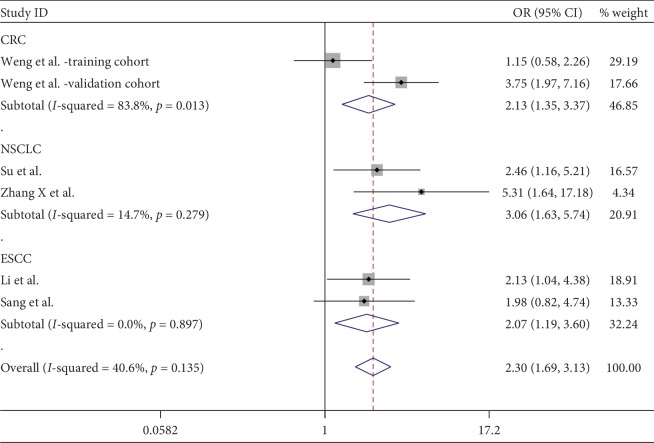
Forest plot for the association between ciRS-7 expression and tumor stage in various cancers.

**Figure 6:**
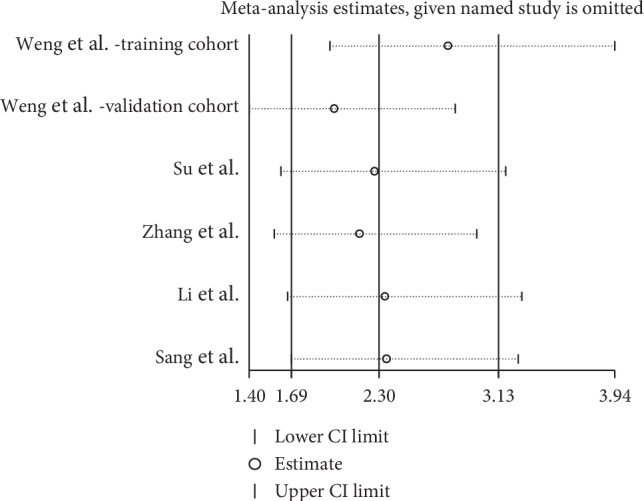
Sensitivity analysis between ciRS-7 expression and clinical stage.

**Figure 7:**
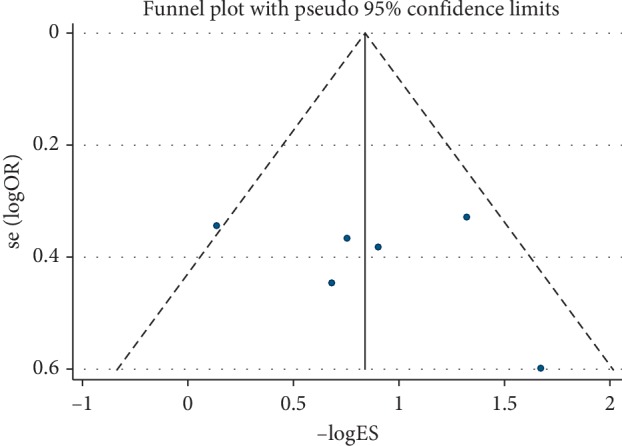
Publication bias of ciRS-7 expression and clinical stage.

6 cohorts from 5 studies comprising 715 patients provided valid data for the correlation between ciRS-7 and clinical stage [19, 22, 24, 29, 30]. Among these 6 cohorts, there were 3 types of cancers (2 CRC, 2 NSCLC, and 2 ESCC). The result showed that an increased ciRS-7 expression was associated with a higher clinical stage (OR = 2.30, 95% CI: 1.69–3.13, *P* < 0.001). Cancer type by subgroup analysis indicated that the positive correlation between ciRS-7 and clinical stage was found in CRC, NSCLC, and ESCC (Figure 5). Significant heterogeneity was not observed in this statistic due to *I*^2^ = 40.6%. Begg's test was performed, and no significant publication bias was found for the clinical stage (*P*=1.000) (Figure 6). Sensitivity analysis was performed through the sequential omission of every single publication, and the results were not significantly altered (Figure 7).

## 4. Discussion

Despite the advancement in treatment modalities, cancer is still one of the leading causes of death worldwide. It is estimated that there will be 22.2 million new cancer cases in 184 countries by 2030 [39]. Therefore, novel biological-specific biomarkers are urgently needed for cancer early detection. Besides prognosis, biomarker is an important priority, which can affect clinical decision-making and the overall results.

Increasing evidence demonstrated that circRNAs are attractive candidates for diagnostic and prognostic biomarkers [40, 41], and among them, ciRS-7 stands out as an oncogenic circRNA at least for a subset of cancer types. Meanwhile, in some studies, ciRS-7 also exerts anti-oncogenic functions [42, 43]. Although researches on ciRS-7 develop fast, the prognostic role of ciRS-7 in cancer is still uncertain. For that reason, the study was conducted, and to our knowledge, no meta-analyses evaluated the correlation of ciRS-7 with the prognosis/clinical stages in numerous cancer patients. In this study, we combined the outcomes of 1,628 patients from 12 available cohorts, indicating that high expression of ciRS-7 was significantly associated with worse survival. In stratified analysis, the results showed that upregulated ciRS-7 predicted worse OS in NSCLC, CRC, and GC. Furthermore, a high level of ciRS-7 was significantly correlated with higher tumor stage in CRC, NSCLC, and ESCC. Sensitivity analysis showed that our results were stable. Importantly, it should be considered that significant heterogeneity was observed in the overall analysis. To find the heterogeneity, a meta-regression was performed and it indicated that sample size might be a contributing factor; yet, there might be other factors that could influence heterogeneity; unfortunately, studies included did not provide individual patient data.

In subgroup analysis, ciRS-7 showed inconsistent prognostic effects in different cancer types. In NSCLC, CRC, and GC, high expression of ciRS-7 was found to be associated with worse prognosis. While in “others” subgroup comprising ESCC, BC, LSCC, and CAA, the number of studies for each cancer type was only one, and the result showed HR = 1.90 (1.00–3.61), *P*=0.05; therefore, we cannot reach a conclusion statistically that ciRS-7 has a role in predicting in this group. More studies are needed for credible conclusion for these four cancer types. The ethnic difference in cancer has been identified and investigated in many studies [44–46]. As is the case with cancer type, no significant association was observed between ciRS-7 expression and Caucasian populations due to small sample size, which may be buttressed by meta-regression. According to the results of meta-regression, we found that sample size is most likely to be the greatest impact on heterogeneity among all factors, because although *P* value of the sample size is 0.066, it is the lowest among all the factors.

To understand the prognostic role of ciRS-7, the regulatory mechanism network of ciRS-7 in cancer needs to be considered. As a competing endogenous RNA, ciRS-7 is ∼1,500 nucleotides in length [47], and the ciRS-7/miR-7 axis should be the major mechanism for the reason that ciRS-7 possesses 73 conventional binding sites [15]. To be noted, many studies indicate a tumor-suppressive role for miR-7, and its downregulation is correlated with poor prognosis in cancer patients [48–50]. Studies have confirmed that miR-7 can inhibit the growth of various types of cancer cells through a variety of key molecular pathways [51, 52]. miR-7 also directly targets insulin-like growth factor 1 receptor (IGF1R) [53], FAK [54], and PAK1 [55], which can inhibit the proliferation, migration, invasion, and epithelial-mesenchymal transition (EMT) of tumor cells. Recent studies show that, as a strong sponge for miR-7, ciRS-7 overexpression is found in GC [23], CRC [21], NSCLC [22], ESCC [30], and CAA [26]. Besides, ciRS-7 can abrogate the tumor-suppressive effect of miR-7 via different cancer-associated signaling pathways including EGFR [21, 22], PTEN/PI3K/AKT [23], NF-*κ*B [24, 25], and IGF1R [21]. Taken together, the prognostic role of ciRS-7 in various cancer could be well explained.

With the closed ring structure, circRNAs are more stable, not easy to be degraded by RNase and high abundance than mRNA [56], which make circRNAs have more advantages in the development and application as a new clinical diagnostic marker. In view of the current researches, there are many prospects for the clinical application of ciRS-7. Recent studies found that circRNAs can be detected in blood and exosomes of cancer patients [57–59]. In the future, it will better reflect its clinical application value, if ciRS-7 can be detected in a noninvasive way. Furthermore, ciRS-7 is expected to become an emerging drug's target. Studies show ciRS-7 overexpression can activate the EGFR pathway, which is one of the most important targets of NSCLC, to induce tumor cell growth [21, 22]. These findings will provide new opportunities for ciRS-7 to be developed as a targeted drug in the future.

Several limitations in this study should be carefully considered. Firstly, the number of included studies was limited which may weaken the power of the results. Secondly, not all different factors were analyzed in this meta-analysis because it was literature-based, and we only got the summarized data rather than individual patient data. Thirdly, some studies had not reported the cutoff values of ciRS-7, and there was no consensus for calculating the cutoff value. Fourthly, we extracted HR and 95% CI from the survival curve rather than directly from the original study for one study, which might impact the results. Finally, although publication biases on OS and clinical stage were not found by Begg's test, there may be an underlying publication bias as studies with positive results could be more likely to be published than those with negative results. Therefore, our meta-analysis still needs further investigation.

In conclusion, this meta-analysis showed that upregulation of ciRS-7 is associated with adverse survival and higher clinical stage in multiple types of cancers. ciRS-7 may be a new star of the future drug target for fighting against tumor growth or used as a biomarker for predicting the prognosis of cancer patients. Larger sample sizes and well-designed studies are needed in the future.

## Figures and Tables

**Table 1:** Characteristics of studies included in this meta-analysis.

Authors	Country	Type of sample	Study design	Type of cancer	Expression of ciRS-7^*∗∗∗*^	Cutoff value	Sample size	NOS score	Follow-up	HR (95% CI) for OS	Detection method	Analysis method
Jiang et al. [26]	China	Tissue	Retrospective	CCA	Upregulation	NR	54	6	NR	1.887 (1.025–3.474)	qRT-PCR	M
Weng et al. Training cohort [19]	China	Tissue	Retrospective	CRC	Upregulation	Median	153	8	3.7y-median	1.8689 (1.0977–3.9023)	qRT-PCR	M
Weng et al. Validation cohort [19]	Japan	Tissue	Retrospective	CRC	Upregulation	Median	165	8	5.1y-median	2.7262 (1.0879–6.8315)	qRT-PCR	M
Tang et al. [21]	China	Tissue	Retrospective	CRC	Upregulation	Median	182	6	NR	1.799 (1.055–3.068)	qRT-PCR	U
Pan et al. Training cohorts [23]	China	Tissue	Prospective	GC	Upregulation	Median	102	6	NR	2.11 (1.1610–3.8348)	qRT-PCR	U
Pan et al. Validation cohort [23]	China	Tissue	Prospective	GC	Upregulation	Median	154	6	NR	2.63 (1.3177–5.2493)	qRT-PCR	U
Su et al. [24]	China	Tissue	Retrospective	NSCLC	Upregulation	Mean	128	6	NR	1.705 (1.02–2.86)	qRT-PCR	M
Zhang et.al. [22]	China	Tissue	Retrospective	NSCLC	Upregulation	Median	60	7	NR	6.132 (2.923–7.556)	qRT-PCR	M
Li et al. [29]	China	Tissue	Retrospective	ESCC	Upregulation	Median	123	7	NR	2.70 (1.55–4.71)	qRT-PCR	U^*∗∗*^
Uhr et al. [27]	Netherlands	Tissue	Retrospective	BC	Upregulation	Median	345	8	91m-median	1.03 (0.90–1.17)	qRT-PCR	U
Yan et al. [18]	China	Tissue	Retrospective	NSCLC	Upregulation	Median	132	8	46m-median	1.575 (1.016–2.440)	qRT-PCR	M
Zhang J et al. [28]	China	Tissue	Retrospective	LSCC	Upregulation	NR	30	6	NR	4.026 (1.144–14.178)	qRT-PCR	U
Sang et al.^*∗*^[30]	China	Tissue	Retrospective	ESCC	Upregulation	Median	86	NR	NR	NR	qRT-PCR	NR

CCA, cholangiocarcinoma; CRC, colorectal cancer; GC, gastric cancer; NSCLC, non-small-cell lung cancer; BC, breast cancer; LSCC, laryngeal squamous cell carcinoma; ESCC, esophageal squamous cell carcinoma; NR, not reported; qRT-PCR, quantitative real-time-PCR; U, univariate analysis; M, multivariate analysis; ^*∗*^the study only reporting the association between ciRS-7 expression and tumor stage, no survival data; ^*∗∗*^HR and corresponding 95% CIs were extracted from the survival curve; ^ *∗∗∗*^compared with the normal tissue.

**Table 2:** Results of subgroup meta-analysis and meta-regression analysis.

Subgroup	No. of studies	No. of patients	Random-effects model		Heterogeneity		Meta-regression *P* value
			HR (95% CI)	*P* value	Ph	*I* ^2^ (%)	
*Cancer type*							0.860
NSCLC	3	320	2.50 (1.07–6.07)	0.035	<0.001	90	
CRC	3	500	1.95 (1.34–2.84)	<0.001	0.735	0	
GC	2	256	2.32 (1.48–3.64)	<0.001	0.636	0	
Others	4	552	1.90 (1.00–3.61)	0.05	<0.001	83.3	

*Ethnicity*							0.111
Asian	11	1283	2.35 (1.17–3.11)	<0.001	0.009	57.6	
Caucasian	1	345	1.03 (0.90–1.17)	0.659			

*Sample size*							0.066
≥100	9	1484	1.83 (1.33–2.50)	<0.001	<0.001	74.8	
<100	3	144	3.60 (1.52–8.52)	0.004	0.012	77.6	

*Analysis method*							0.772
Univariable	6	936	2.00 (1.23–3.25)	0.005	<0.001	81.8	
Multivariable	6	692	2.32 (1.44–3.76)	0.001	0.001	76.7	

*Cut-off value*							0.208
Median	9	1416	2.19 (1.39–3.45)	0.001	<0.001	88.9	
Mean and others	3	212	1.91 (1.31–2.79)	0.001	0.464	0	

*Study design*							0.924
Retrospective	10	1372	2.14 (1.41–3.26)	<0.001	<0.001	87.3	
Prospective	2	256	2.32 (1.48–3.64)	<0.001	0.636	0	

NSCLC, non-small-cell lung cancer; CRC, colorectal cancer; GC, gastric cancer; Ph, the *P* value of *Q* test for heterogeneity test.

## References

[1] Sanger H. L., Klotz G., Riesner D., Gross H. J., Kleinschmidt A. K. (1976). Viroids are single-stranded covalently closed circular RNA molecules existing as highly base-paired rod-like structures. *Proceedings of the National Academy of Sciences*.

[2] Kolakofsky D. (1976). Isolation and characterization of Sendai virus DI-RNAs. *Cell*.

[3] Memczak S., Jens M., Elefsinioti A. (2013). Circular RNAs are a large class of animal RNAs with regulatory potency. *Nature*.

[4] Szabo L., Salzman J. (2016). Detecting circular RNAs: bioinformatic and experimental challenges. *Nature Reviews Genetics*.

[5] Barbagallo D., Caponnetto A., Brex D. (2019). CircSMARCA5 regulates VEGFA mRNA splicing and angiogenesis in glioblastoma multiforme through the binding of SRSF1. *Cancers*.

[6] Bach D.-H., Lee S. K., Sood A. K. (2019). Circular RNAs in cancer. *Molecular Therapy-Nucleic Acids*.

[7] Wan L., Zhang L., Fan K., Cheng Z. X., Sun Q. C., Wang J. J. (2016). Circular RNA-ITCH suppresses lung cancer proliferation via inhibiting the wnt/*β*-catenin pathway. *BioMed Research International*.

[8] Zheng H., Chen T., Li C. (2019). A circular RNA hsa_circ_0079929 inhibits tumor growth in hepatocellular carcinoma. *Cancer Management and Research*.

[9] Gao D., Qi X., Zhang X., Fang K., Guo Z., Li L. (2019). hsa_circRNA_0006528 as a competing endogenous RNA promotes human breast cancer progression by sponging miR-7-5p and activating the MAPK/ERK signaling pathway. *Molecular Carcinogenesis*.

[10] Li P., Chen H., Chen S. (2017). Circular RNA 0000096 affects cell growth and migration in gastric cancer. *British Journal of Cancer*.

[11] Yang C., Yuan W., Yang X. (2018). Circular RNA circ-ITCH inhibits bladder cancer progression by sponging miR-17/miR-224 and regulating p21, PTEN expression. *Molecular Cancer*.

[12] Liang H. F., Zhang X. Z., Liu B. G., Jia G. T., Li W. L. (2017). Circular RNA circ-ABCB10 promotes breast cancer proliferation and progression through sponging miR-1271. *American Journal of Cancer Research*.

[13] Hsiao K.-Y., Lin Y.-C., Gupta S. K. (2017). Noncoding effects of circular RNA CCDC66 promote colon cancer growth and metastasis. *Cancer Research*.

[14] Ma X., Yang X., Bao W. (2018). Circular RNA circMAN2B2 facilitates lung cancer cell proliferation and invasion via miR-1275/FOXK1 axis. *Biochemical and Biophysical Research Communications*.

[15] Hansen T. B., Jensen T. I., Clausen B. H. (2013). Natural RNA circles function as efficient microRNA sponges. *Nature*.

[16] Xu H., Guo S., Li W., Yu P. (2015). The circular RNA Cdr1as, via miR-7 and its targets, regulates insulin transcription and secretion in islet cells. *Scientific Reports*.

[17] Vo J. N., Cieslik M., Zhang Y. (2019). The landscape of circular RNA in cancer. *Cell*.

[18] Yan B., Zhang W., Mao X. W., Jiang L. Y. (2018). Circular RNA ciRS-7 correlates with advance disease and poor prognosis, and its down-regulation inhibits cells proliferation while induces cells apoptosis in non-small cell lung cancer. *European Review for Medical and Pharmacological Sciences*.

[19] Weng W., Wei Q., Toden S. (2017). Circular RNA ciRS-7-A promising prognostic biomarker and a potential therapeutic target in colorectal cancer. *Clinical Cancer Research*.

[20] Yu L., Gong X., Sun L., Zhou Q., Lu B., Zhu L. (2016). The circular RNA Cdr1as act as an oncogene in hepatocellular carcinoma through targeting miR-7 expression. *PLoS One*.

[21] Tang W., Ji M., He G. (2017). Silencing CDR1as inhibits colorectal cancer progression through regulating microRNA-7. *OncoTargets and Therapy*.

[22] Zhang X., Yang D., Wei Y. (2018). Overexpressed CDR1as functions as an oncogene to promote the tumor progression via miR-7 in non-small-cell lung cancer. *OncoTargets and Therapy*.

[23] Pan H., Li T., Jiang Y. (2018). Overexpression of circular RNA ciRS-7 abrogates the tumor suppressive effect of miR-7 on gastric cancer via PTEN/PI3K/AKT signaling pathway. *Journal of Cellular Biochemistry*.

[24] Su C., Han Y., Zhang H. (2018). CiRS-7 targeting miR-7 modulates the progression of non-small cell lung cancer in a manner dependent on NF-*κ*B signalling. *Journal of Cellular and Molecular Medicine*.

[25] Huang H., Wei L., Qin T., Yang N., Li Z., Xu Z. (2019). Circular RNA ciRS-7 triggers the migration and invasion of esophageal squamous cell carcinoma via miR-7/KLF4 and NF-*κ*B signals. *Cancer Biology & Therapy*.

[26] Jiang X. M., Li Z. L., Li J. L. (2018). A novel prognostic biomarker for cholangiocarcinoma: circRNA Cdr1as. *European Review for Medical and Pharmacological Sciences*.

[27] Uhr K., Sieuwerts A. M., de Weerd V. (2018). Association of microRNA-7 and its binding partner CDR1-AS with the prognosis and prediction of 1(st)-line tamoxifen therapy in breast cancer. *Scientific Reports*.

[28] Zhang J., Hu H., Zhao Y., Zhao Y. (2018). CDR1as is overexpressed in laryngeal squamous cell carcinoma to promote the tumour’s progression via miR-7 signals. *Cell Proliferation*.

[29] Li R.-C., Ke S., Meng F.-F. (2018). CiRS-7 promotes growth and metastasis of esophageal squamous cell carcinoma via regulation of miR-7/HOXB13. *Cell Death & Disease*.

[30] Sang M., Meng L., Sang Y. (2018). Circular RNA ciRS-7 accelerates ESCC progression through acting as a miR-876-5p sponge to enhance MAGE-A family expression. *Cancer Letters*.

[31] Moher D., Liberati A., Tetzlaff J., Altman D. G. (2009). Preferred reporting items for systematic reviews and meta-analyses: the PRISMA statement. *BMJ*.

[32] Stang A. (2010). Critical evaluation of the Newcastle-Ottawa scale for the assessment of the quality of nonrandomized studies in meta-analyses. *European Journal of Epidemiology*.

[33] Xu L., Zhang Y., Tang J. (2019). The prognostic value and regulatory mechanisms of microRNA-145 in various tumors: a systematic review and meta-analysis of 50 studies. *Cancer Epidemiology Biomarkers & Prevention*.

[34] Tierney J. F., Stewart L. A., Ghersi D., Burdett S., Sydes M. R. (2007). Practical methods for incorporating summary time-to-event data into meta-analysis. *Trials*.

[35] Higgins J. P. T., Thompson S. G. (2002). Quantifying heterogeneity in a meta-analysis. *Statistics in Medicine*.

[36] DerSimonian R., Laird N. (1986). Meta-analysis in clinical trials. *Controlled Clinical Trials*.

[37] Mantel N., Haenszel W. (1959). Statistical aspects of the analysis of data from retrospective studies of disease. *Journal of the National Cancer Institute*.

[38] Begg C. B., Mazumdar M. (1994). Operating characteristics of a rank correlation test for publication bias. *Biometrics*.

[39] Bray F., Jemal A., Grey N., Ferlay J., Forman D. (2012). Global cancer transitions according to the Human Development Index (2008–2030): a population-based study. *The Lancet Oncology*.

[40] Huang X., Zhang W., Shao Z. (2019). Prognostic and diagnostic significance of circRNAs expression in lung cancer. *Journal of Cellular Physiology*.

[41] Li X., He M., Guo J., Cao T. (2019). Upregulation of circular RNA circ-ERBB2 predicts unfavorable prognosis and facilitates the progression of gastric cancer via miR-503/CACUL1 and miR-637/MMP-19 signaling. *Biochemical and Biophysical Research Communications*.

[42] Li P., Yang X., Yuan W. (2018). CircRNA-Cdr1as exerts anti-oncogenic functions in bladder cancer by sponging MicroRNA-135a. *Cellular Physiology and Biochemistry*.

[43] Barbagallo D., Condorelli A., Ragusa M. (2016). Dysregulated miR-671-5p/CDR1-AS/CDR1/VSNL1 axis is involved in glioblastoma multiforme. *Oncotarget*.

[44] Soo R. A., Loh M., Mok T. S. (2011). Ethnic differences in survival outcome in patients with advanced stage non-small cell lung cancer: results of a meta-analysis of randomized controlled trials. *Journal of Thoracic Oncology*.

[45] Theuer C. P., Kurosaki T., Ziogas A., Butler J., Anton-Culver H. (2000). Asian patients with gastric carcinoma in the United States exhibit unique clinical features and superior overall and cancer specific survival rates. *Cancer*.

[46] Limkin E. J., Blanchard P. (2019). Does east meet west? Towards a unified vision of the management of nasopharyngeal carcinoma. *The British Journal of Radiology*.

[47] Peng L., Yuan X. Q., Li G. C. (2015). The emerging landscape of circular RNA ciRS-7 in cancer. *Oncology Reports*.

[48] Cheng M.-W., Shen Z.-T., Hu G.-Y., Luo L.-G. (2017). Prognostic significance of microRNA-7 and its roles in the regulation of cisplatin resistance in lung adenocarcinoma. *Cellular Physiology and Biochemistry*.

[49] Nagano Y., Toiyama Y., Okugawa Y. (2016). MicroRNA-7 is associated with malignant potential and poor prognosis in human colorectal cancer. *Anticancer Research*.

[50] Suto T., Yokobori T., Yajima R. (2015). MicroRNA-7expression in colorectal cancer is associated with poor prognosis and regulates cetuximab sensitivity viaEGFRregulation. *Carcinogenesis*.

[51] Horsham J., Kalinowski F., Epis M., Ganda C., Brown R., Leedman P. (2015). Clinical potential of microRNA-7 in cancer. *Journal of Clinical Medicine*.

[52] Ye T., Yang M., Huang D. (2019). MicroRNA-7 as a potential therapeutic target for aberrant NF-*κ*B-driven distant metastasis of gastric cancer. *Journal of Experimental & Clinical Cancer Research*.

[53] Jiang L., Liu X., Chen Z. (2010). MicroRNA-7 targets IGF1R (insulin-like growth factor 1 receptor) in tongue squamous cell carcinoma cells. *Biochemical Journal*.

[54] Kong X., Li G., Yuan Y. (2012). MicroRNA-7 inhibits epithelial-to-mesenchymal transition and metastasis of breast cancer cells via targeting FAK expression. *PLoS One*.

[55] Reddy S. D. N., Ohshiro K., Rayala S. K., Kumar R. (2008). MicroRNA-7, a homeobox D10 target, inhibits p21-activated kinase 1 and regulates its functions. *Cancer Research*.

[56] Jeck W. R., Sorrentino J. A., Wang K. (2013). Circular RNAs are abundant, conserved, and associated with ALU repeats. *RNA*.

[57] Yin W.-B., Yan M.-G., Fang X., Guo J.-J., Xiong W., Zhang R.-P. (2018). Circulating circular RNA hsa_circ_0001785 acts as a diagnostic biomarker for breast cancer detection. *Clinica Chimica Acta*.

[58] Li P., Chen S., Chen H. (2015). Using circular RNA as a novel type of biomarker in the screening of gastric cancer. *Clinica Chimica Acta*.

[59] Fanale D., Taverna S., Russo A., Bazan V. (2018). Circular RNA in exosomes. *Advances in Experimental Medicine and Biology*.

